# Intramuscular tendon length in agonist–antagonist myoneural interface components in transtibial amputation: An anatomic study

**DOI:** 10.1111/joa.14250

**Published:** 2025-05-09

**Authors:** Viktoria Witowski, Lisa Lorbeer, Laura Schmid, Benedict Wilhelmi, Victor A. Hoursch, Matthew J. Carty, Hugh M. Herr, Roland Blumer, Massimo Sartori, Utku Ş. Yavuz, Corey L. Sullivan, Stephan Sehmisch, Andreas Schmiedl, Jennifer Ernst

**Affiliations:** ^1^ Hannover Medical School Department of Trauma Surgery Hannover Germany; ^2^ Institute for Modelling and Simulation of Biomechanical Systems University of Stuttgart Stuttgart Germany; ^3^ Division of Plastic Surgery Brigham & Women's Hospital Boston Massachusetts USA; ^4^ Massachusetts Institute of Technology Center for Extreme Bionics Cambridge Massachusetts USA; ^5^ Center of Anatomy and Cell Biology, Division of Anatomy, Medical Image Cluster Medical University Vienna Vienna Austria; ^6^ Department of Biomechanical Engineering University of Twente Enschede Netherlands; ^7^ Biomedical Signals and Systems University of Twente Enschede Netherlands; ^8^ Hannover Medical School Institute of Functional and Applied Anatomy Hannover Germany; ^9^ Department of Trauma Surgery, Orthopaedics and Plastic Surgery University of Göttingen Göttingen Germany

**Keywords:** agonist–antagonist muscle strain, agonist–antagonist myoneural interface, anatomy, functional limb restoration, lower extremity, mechanoneural interfaces, muscle architecture, muscle–tendon length, proprioception

## Abstract

Understanding the role of tendons in muscle function and proprioception is crucial for enhancing amputation surgery. Muscle spindles and Golgi tendon organs provide essential feedback for muscle control. Preservation of tendon function in amputation surgery and the development of the agonist–antagonist myoneural interface (AMI) have shown promising results restoring muscle–tendon proprioception and in improving prosthetic control. However, challenges remain in constructing AMI due to anatomical limitations in residual limbs. A total of 25 lower legs from fresh‐frozen human Caucasian donors were dissected, and the muscles relevant to the AMI technique, such as the gastrocnemius complex, the tibialis posterior, the tibialis anterior, and the peroneus longus, were analyzed. Demographic and anthropometric measurements, muscle preparation and weight, markings, imaging, and statistical analysis methods were described in detail. In all muscles examined, the intramuscular course of the tendon extended over more than 75% of the distal muscle belly. The muscle belly length of the peroneus longus muscle and the medial head of the gastrocnemius muscle showed a significant positive correlation with the weight and height of the donors. There were no significant correlations between the ratio of the intramuscular course of the tendon to muscle belly length and the weight or height of the donor. The AMI technique can enhance proprioceptive feedback for transtibial amputees wearing prostheses. The study indicates that gender does not impact muscle characteristics, but weight and height show correlations. These results offer valuable insights into muscle anatomy for informing future research on the functional effects of AMI and prosthetic limb design.

## Graphical abstract

Interestingly, this study shows that tendons spanned over 75% of the distal muscle belly in all muscles, crucial for the innovative tibiotalar agonist–antagonist myoneural interface (AMI) construction. AMI simulates natural muscle–tendon interaction in amputees, improving prosthetic control despite anatomical constraints. Leg length was compared to muscle belly length (MBL) and intramuscular tendon length (LICT). The gastrocnemius (MG/LG) had the longest LICT, while the peroneus longus (PL) had the shortest.

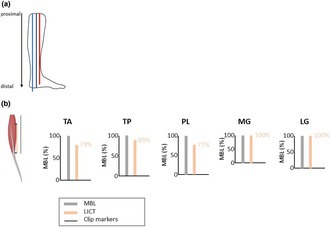

## 1 INTRODUCTION

The muscle–tendon units of the human body have a variable and complex design (Bojsen‐Møller & Magnusson, 2019). Proprioception, sometimes referred to as the “sixth sense,” integrates the signals for force, movement, and position to describe the internal perception of the orientation of the body in space (Moon et al., 2021). In mammalian skeletal muscle, Golgi tendon organs (GTO) and muscle spindles (MS) monitor changes in muscle length and tension (Jami, 1992; Moon et al., 2021; Shadrach et al., 2021) and then transmit that information from the periphery to the spinal cord via afferent axons. There, the information is in part consciously perceived by the sensory cortex and partly unconsciously by the cerebellum. MS work through monosynaptic transmission while GTOs' afferent feedback is mediated through disynaptic inhibitory interneurons (Dietz & Filli, 2022; Santuz et al., 2022). Typically, GTOs are located at the muscle–tendinous junctions and process information about contraction and tension while MS are localized in the muscle belly to measure changes in length and speed of fire in response to stretch (Blum et al., 2017; Horváth et al., 2023; Jami, 1992; Proske & Gandevia, 2012). Tendons transmit force from muscle to bone and store energy (Kirkendall & Garrett, 1997).

However, tendons are bradytrophic tissues with limited or no direct blood supply and prolonged healing time under pathological conditions (Tempfer et al., 2015; Zantop et al., 2003). This might explain why for centuries, the bedrock of amputation surgery has been to transect and remove tendons together with other non‐viable tissue. Recently, novel amputation techniques have sought to preserve or restore function (Chiao et al., 2023; Clites, Herr, et al., 2018, Clites, Carty, et al., 2018; Herr et al., 2021). The utilization of tendons in such procedures has resulted in innovative attempts to exploit their role in proprioception and neural signaling for prosthetic control strategies. Within anatomically intact limbs, mechanically coupled antagonistic muscles spanning an articular joint enable afferent signaling from MS and GTOs corresponding to balanced limb movements and peripheral proprioceptive feedback through agonist–antagonist muscle strain (AMS) (Clites, Herr, et al., 2018; Proske & Gandevia, 2012; Song et al., 2022; Srinivasan et al., 2020). In standard amputation procedures, the connection and communication of the agonist and antagonist muscles become disrupted, compromising function and sensory feedback. Recently, the agonist–antagonist myoneural interface (AMI) was developed to restore AMS for persons with amputation and provide muscle–tendon proprioception and improved neuroprosthetic control (Song et al., 2022). Published outcomes have shown promising, clinically relevant results (Berger et al., 2023; Chiao et al., 2023; Clites, Herr, et al., 2018; Sullivan et al., 2023).

**FIGURE 1:**
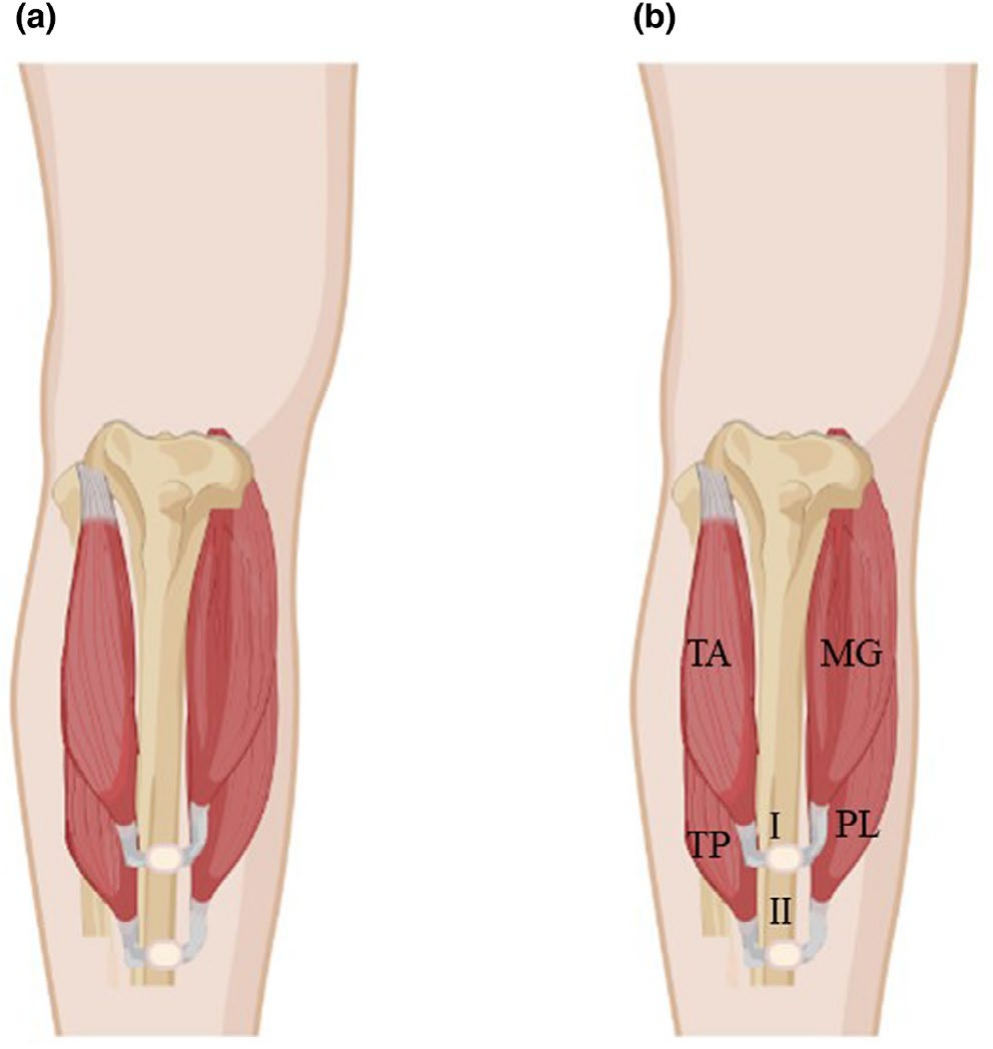
Schematic illustration of component muscles in a standard transtibial AMI procedure. In the typical configuration, two AMIs (I, II) are constructed via coaptation of the TA and MG (I) as well as the TP and the PL (II). MG, medial gastrocnemius; PL, peroneus longus; TA, tibialis anterior; TP, tibialis posterior. Created with BioRender.com.

The surgical feasibility and clinical functionality of AMI constructs require the presence of tendons and expression of MS and GTOs at the level of the amputation. Due to anatomic limitations present in the residual limb, however, AMI construction often requires coaptation of agonist and antagonist muscles at a level proximal to their visible musculotendinous junctions (Figure 1). It is assumed that the portion of the muscle that remains following AMI construction contains sufficient residual intramuscular tendon to elicit appropriate activation of MS and GTOs to recapitulate native proprioceptive pathways; however, the validity of this assumption has yet to be verified. Stated another way, even though the arrangement of muscles and tendons has been studied in detail by anatomists, surgeons, and biomechanists, there is little knowledge about the intramuscular course of extremity muscles' tendons (Dziedzic et al., 2014; Heron & Richmond, 1993; Wilson & Lichtwark, 2011).

Thus, the purpose of this study was to investigate the morphology and anatomical relationship of muscles and their intramuscular tendons relevant to AMI construction in a transtibial amputation scenario. It is expected that the results of this investigation may serve as a reference guide for surgical planning and enhance our anatomic understanding of the neurophysiological benefits witnessed to date in AMI construction.

## 2 METHODS AND MATERIALS

### 2.1 Materials

Gastrocnemius complex (GC), tibialis posterior (TP), tibialis anterior (TA), and peroneus longus muscles (PL) from 25 fresh frozen human Caucasian donors were prepared.

### 2.2 Demographics and anthropomorphic measurements

Age, weight, height, leg length, and BMI of the human body donors were collected in a tabular sheet (Table 1). The leg length (cm) was measured from the proximal border of the tibial plateau to the medial malleolus with the tibiotalar joint at a 90‐degree angle.

**TABLE 1:** Demographics and anthropometric data of donors (*n* = 25).

Parameter (*n* = 25)	Mean (SD)			Range (maximum—minimum)		
	All	Female (*n* = 14)	Male (*n* = 11)	All	Female (*n* = 14)	Male (*n* = 11)
Age (years)	83.40 ± 8.02	84 ± 8	83 ± 8.	97–61	97–69	91–61
Weight (kg)	63.61 ± 19.43	58.69 ± 19.22	70.00 ± 18.71	100–35	85–35	100–45
Height (cm)	172 ± 0	165 ± 4	181 ± 9	190–157	170–157	190–163
Leg length (cm)	46 ± 3	44 ± 1.40	48 ± 3	52–41	46–41	52–43
BMI (kg/m^2^)	21 ± 6	21 ± 7	21 ± 5	30–13	30–13	28–14

*Note*: Given are means and standard deviations (SD) and the maximum and minimum values for all samples as well as split in females and males. Length (cm) = tibia plateau to the plantar heel pad with the tibiotalar joint at 90‐degree angle.

### 2.3 Dissection and muscle weight

Dissection of the leg began with the identification and isolation of the medial gastrocnemius (MG) and the lateral gastrocnemius (LG) muscles, followed by the tibialis posterior (TP), tibialis anterior (TA), and peroneus longus (PL) muscles (Table 2). The muscles were thoroughly isolated from their bony origins and insertions to accurately reflect the complete length of the muscle bellies and their corresponding tendons in situ. Attached subcutaneous fat was removed, and each dissected muscle (including tendon) was weighed (total muscle weight, TMW) (MC1 Laboratory LC2200 S, Sartorius AG, Germany).

**TABLE 2:** Characteristics of the dissected leg muscles.

	Mean (SD) LICT (cm)	Mean (SD) MBL (cm)	LICT: MBL ratio (SD)	Mean (SD) TMW (g)
TA (*n* = 25)	21.63 (2.80)	27.34 (2.89)	0.79 (0.07)	64.77 (24.00)
TP (*n* = 24)	22.18 (3.65)	24.91 (3.29)	0.89 (0.10)	39.88 (18.63)
PL (*n* = 25)	18.37 (4.64)	23.97 (5.27)	0.77 (0.12)	40.49 (26.55)
MG (*n* = 25)	16.92 (2.20)	16.92 (2.20)	1.00 (0.00)	171.07 (67.91)
LG (*n* = 25)	19.91 (2.91)	19.93 (2.92)	1.00 (0.00)	

*Note*: The LICT (cm), the MBL (cm), the LICT:MBL ratio, as well as the TMW (g) are shown as means with SD. The MG and LG were weighed together.

Abbreviations: LG, lateral gastrocnemius; LICT, length of the intramuscular course of the tendon; MBL, muscle belly length; MG, medial gastrocnemius muscle; PL, peroneus longus; SD, standard deviation.; TA, tibialis anterior; TMW, total muscle weight; TP, tibialis posterior.

### 2.4 Markings and imaging

The intramuscular part of the tendon of each harvested muscle was dissected carefully from distal to proximal. The distal entry point of the tendon into the muscle belly and the most proximal still macroscopically visible tendon in each muscle belly was marked by vessel clips (B. Braun SE, Germany).

**FIGURE 2:**
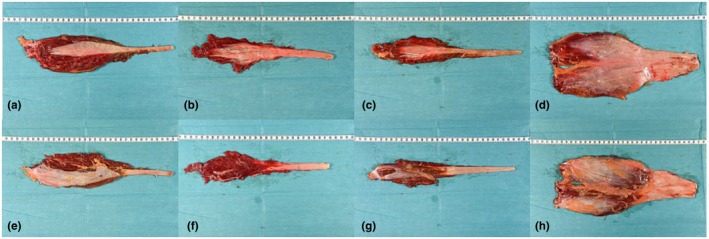
Exemplary dissected leg muscles of a fresh frozen human body donor. The upper row (a–d) illustrates the muscles from the ventral side and the lower row (e–h) from the dorsal side. The distal entry point of the tendon into the muscle belly to the most proximal muscular portion was measured and scored as MBL. The most distal entry point of the tendon and the most proximal macroscopically visible tendon in each muscle belly were measured and characterized as LICT. The LICT was measured from the ventral side and the MBL from the dorsal side. From left to right for both the upper and lower rows: TA (a, e), TP (b, f), PL (c, g), and GC (d, h). GC, gastrocnemius complex; LICT, length of the intramuscular course of the tendon; MBL, muscle belly length; PL, peroneus longus; TA, tibialis anterior; TP, tibialis posterior.

The dissected and labeled muscles of one muscle group were placed on a green background (Figure 2). A centimeter measuring tape (Co‐med GmbH & Co. KG, Germany) was added before taking pictures from the ventral and dorsal aspects of every single harvested muscle. The pictures were imported into the GNU Image Manipulation Program (GIMP; www.gimp.org, version 2.8), an open‐source software. Muscle belly length (MBL) and the length of the intramuscular course of the tendon (LICT) were calculated using ImageJ software (imagej.nih.gov/ij, version Java 8, 2015) by measuring the distance between the clips using the wand (tracing) tool, followed by the “analyze” and “measure” functions.

### 2.5 Statistical analysis

We calculated the arithmetic mean and standard deviation (SD) for each measured parameter and each muscle. We investigated if there were significant differences between genders for the demographic and anthropometric data and the LICT:MBL ratio. We used a Shapiro–Wilk test to determine if the data was normally distributed. For normally distributed data, we utilized a two‐sample *t*‐test to compare the groups and a Wilcoxon rank sum test for non‐normally distributed data. We correlated the MBL and the TMW to the human body donors' weight, height, and gender. Furthermore, LICT:MBL ratio was compared to the human body donors' weight, height, and gender. Correlations were quantified using the Pearson correlation coefficient. In all tests, the significance level was set to *p* < 0.05. All statistical analyses were performed with MATLAB R2021a.

## 3 RESULTS

### 3.1 Demographics and anthropomorphic measurements

One hundred total muscles from 25 fresh‐frozen human legs were harvested (Figure S2). The donor mean age was 83.40 ± 8.02 years at the time of death. Donor mean height was 172 ± 10 cm overall; mean female height was 165 ± 4 cm and mean male height was 181 ± 9 cm. Donor mean BMI was 21 ± 6 kg/m^2^; mean female BMI was 21 ± 7 kg/m^2^ and mean male BMI was 21 ± 5 kg/m^2^. The male: female ratio was 1.3 and right leg: left leg ratio was 1.5 (Table 1). Mean length from the tibial plateau to the plantar heel pad with the tibiotalar joint at 90‐degree angle to the tibia was 46 ± 3 cm overall, with the mean female leg length being 44 ± 1 cm and the mean male leg length being 48 ± 3 cm (Table 1). Height (*p* = 0.002) and leg length (*p* < 0.001) were significantly shorter in females than in males.

### 3.2 Characterization of the leg muscles and their tendon–muscle relationship

**FIGURE 3:**
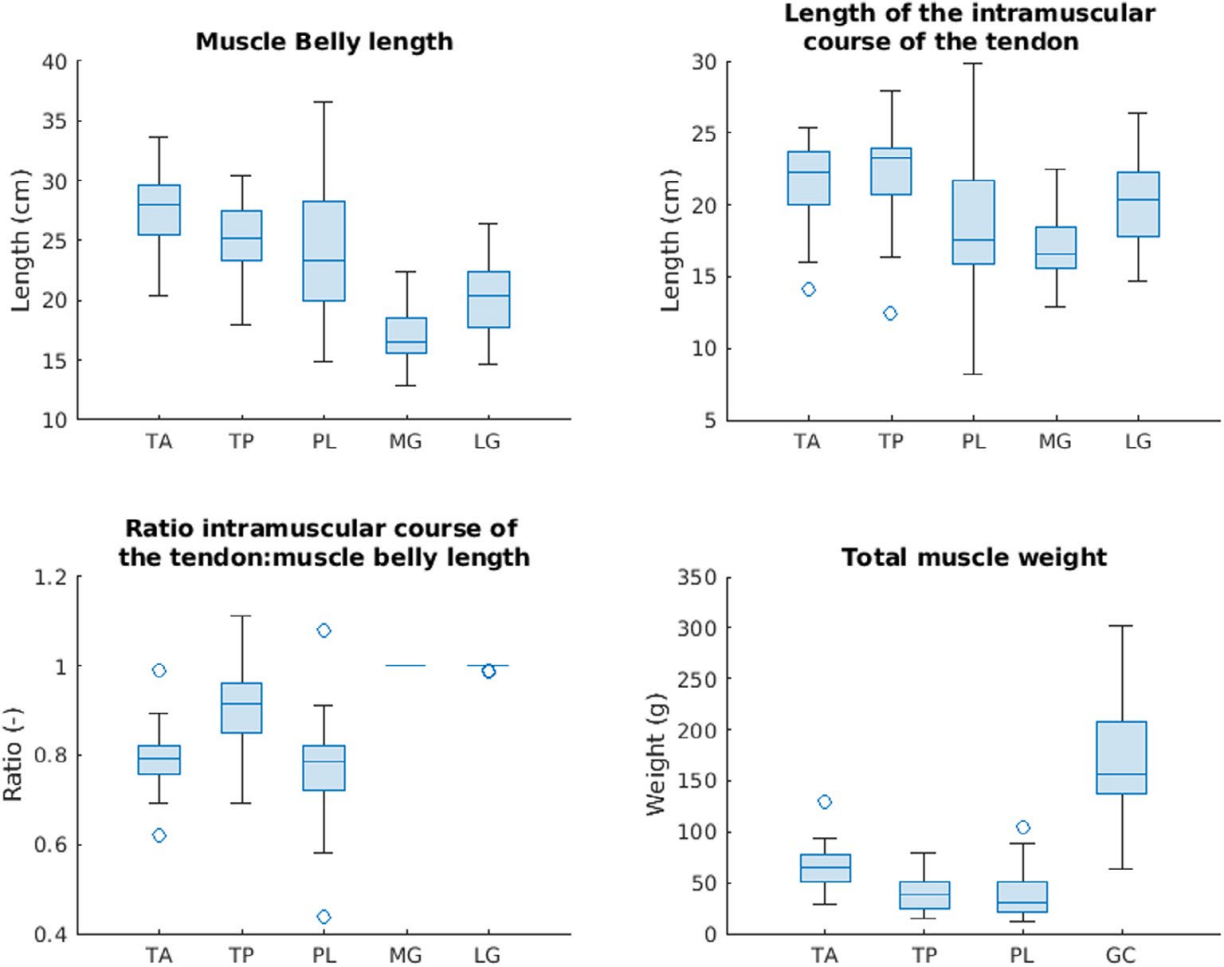
Boxplots describing the distribution of the analyzed parameters of the donor legs. The MBL (cm), the LICT (cm), the ratio of the intramuscular course of the tendon: muscle belly length (cm) as well as the TMW (g) are shown as boxplots. Each boxplot displays the median, the lower and upper quartiles, any outliers (computed using the interquartile range, outliers are marked with a circle (O) on the boxplot), and the minimum and maximum values that are not outliers. The MG and LG were weighted together. LG, lateral head gastrocnemius muscle; LICT, length of the intramuscular course of the tendon; MBL, muscle belly length; MG, medial head gastrocnemius muscle; PL, peroneus longus muscle; TA, tibialis anterior muscle; TMW, total muscle weight; TP, tibialis posterior muscle.

**FIGURE 4:**
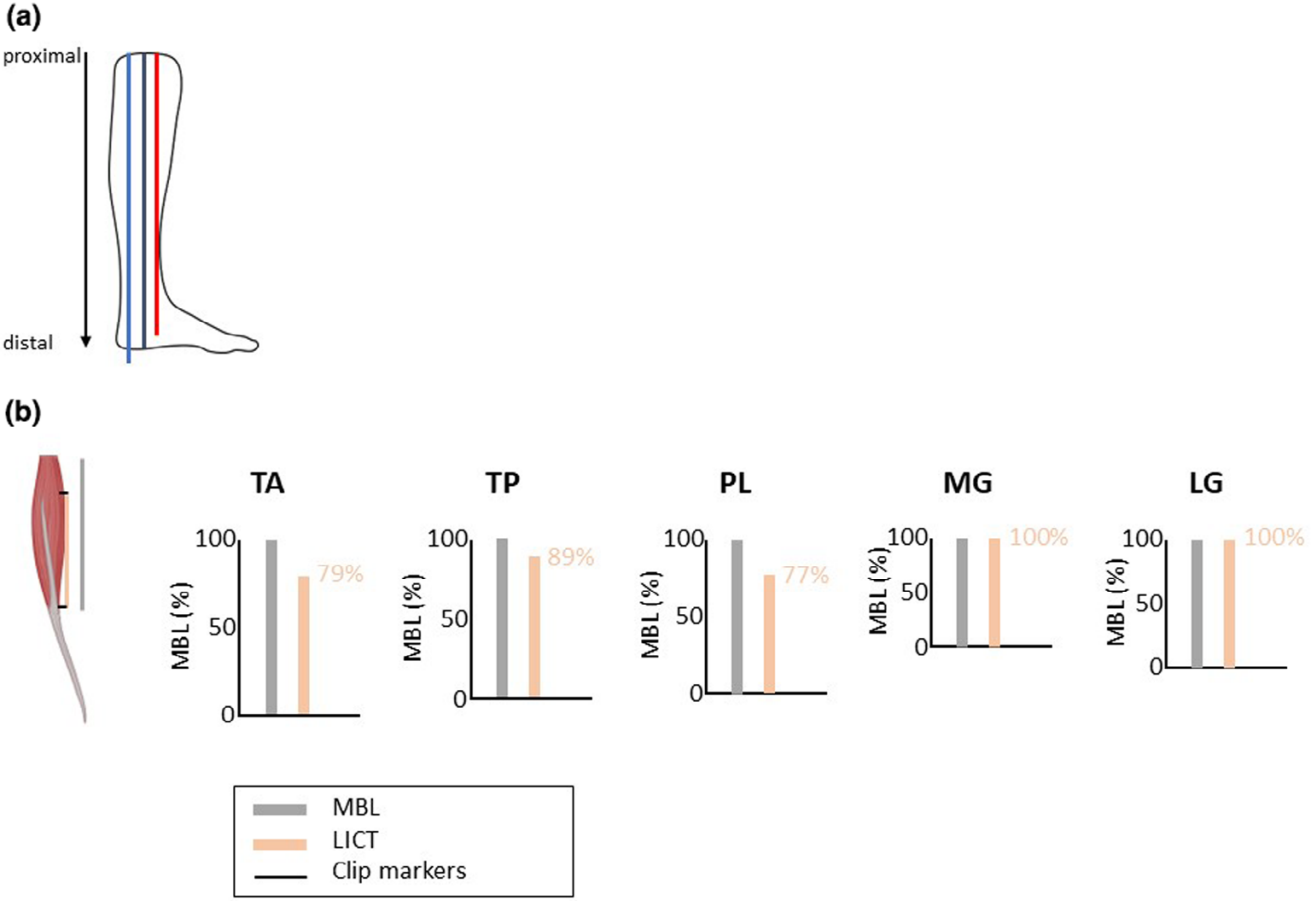
Illustration of the leg compared to MBL and LICT for each dissected leg muscle. The mean leg length of all human body donors was 45.77 cm (black bar); males were 48.20 cm (blue bar) and females were 43.86 cm (red bar) (a). The grey bars indicate the MBL (cm), the orange bar indicates the LICT (cm) for the dissected leg muscles (b). In relation to the MBL, the MG and LG have the longest LICT as is it covers the entire length of the dorsal site of the muscle belly. The PL has the shortest LICT relative to the MBL. For all investigated muscles, the tendon spans more than 75% of the distal muscle belly. Macroscopic visible tendinous tissue spans 100% of the MG and LG muscle belly. Within AMI surgery, the pure tendinous portion of the LG or MG is used for tibiotalar AMI construction (Berger et al., 2023). Macroscopic visible tendinous tissue spans 77% of the PL, 89% of the TP, and 79% of the TA. LG, lateral gastrocnemius; LICT, length of the intramuscular course of the tendon; MBL, muscle belly length; MG, medial gastrocnemius; PL, peroneus longus; TA, tibialis anterior; TP, tibialis posterior.

The TMW, MBL, LICT, and the ratio of tendon and muscle length were calculated for each muscle (Table 2, Figures 3 and 4).

On average, the muscle with the longest MBL was the TA (27.34 ± 2.89 cm), while the shortest was the MG (16.92 ± 2.2 cm). The MG was also the only muscle that was significantly longer in males than in females (*p* = 0.02) (Figure 4). The longest intramuscular course of the tendon was in the TP (22.18 ± 3.65 cm) and the shortest was in the MG (16.92 ± 2.2 cm). In relation to the MBL, the MG and LG demonstrated the longest intramuscular courses, as they covered the entire length of the dorsal site of the muscle bellies. The PL had the shortest intramuscular course of the tendon relative to the muscle belly length. For all investigated muscles, the tendon spanned more than 75% of the distal muscle belly. Macroscopically visible tendinous tissue spanned 100% of the MG and LG muscle bellies. Macroscopically visible tendinous tissue spanned the distal 77% of the PL, 89% of the TP, and 79% of the TA (Figure S4).

**FIGURE 5:**
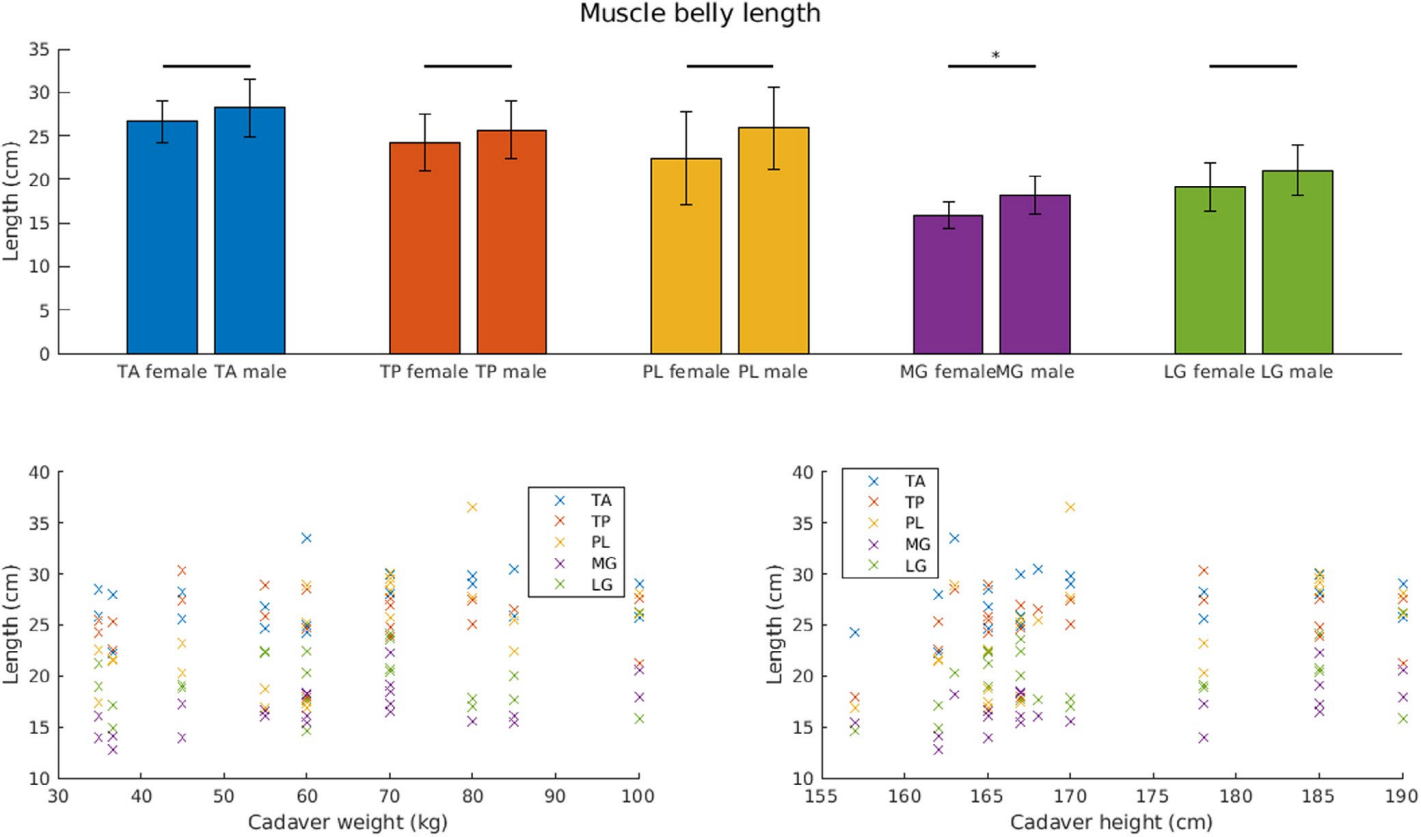
Correlation of MBL to donor weight and height. Top row: MBL for females and males. Significant differences are marked with an asterisk (*). Bottom row: MBL over cadaver donors body weight (left) and donors' height (right). We correlated the MBL to donors' weight and height. The MBL of PL and MG show a significant positive correlation with both donors' weight and height (PL weight: correlation coefficient *ρ* = 0.55, *p* < 0.01; PL height: *ρ* = 0.53, *p* < 0.01; MG weight: *ρ* = 0.52, *p* = 0.01; MG height: *ρ* = 0.59, *p* < 0.01). LG, lateral gastrocnemius; MBL, muscle belly length; MG, medial gastrocnemius; PL, peroneus longus; TA, tibialis anterior; TP, tibialis posterior.

**FIGURE 6:**
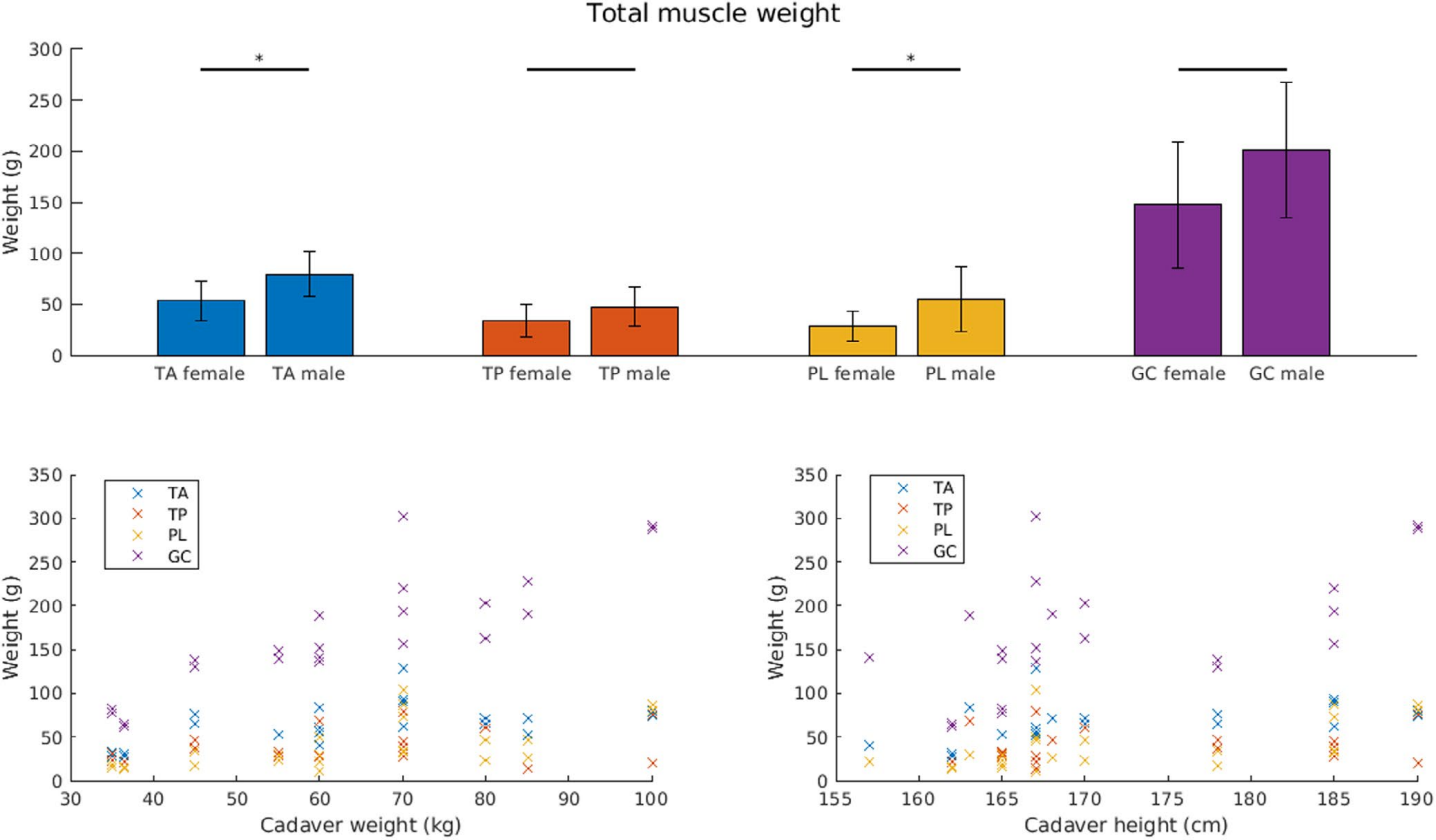
Correlation of TMW to donor weight and height. Top row: TMW for females and males. Significant differences are marked with a star (*). Bottom row: TMW over donor weight (left) and donor height (right). We correlated the TMW to both the donors' weight and height. The TMW of all muscles but TP is significantly positively related to both cadaver donors body weight and height (TA‐weight: *ρ* = 0.55, *p* < 0.01; TA height: *ρ* = 0.49, *p* = 0.02; PL weight: *ρ* = 0.63, *p* < 0.01; PL height: *ρ* = 0.58, *p* < 0.01; GC weight: *ρ* = 0.86, *p* < 0.01; GC height, *ρ* = 0.52, *p* = 0.01). GC, gastrocnemius complex; PL, peroneus longus; TA, tibialis anterior; TMW, total muscle weight; TP, tibialis posterior.

The TMW of the TP was the lowest, with a mean of 39.88 ± 18.63 g. The MG and LG muscles were weighted together; therefore, the average of those muscles was the most (171.07 ± 67.91 g). The TA and PL were significantly heavier in males than in females (*p* = 0.02, *p* = 0.03) (Figure 5). The ratio of macroscopically visible tendon to the MBL did not differ significantly between females and males (Figure 6).

**FIGURE 7:**
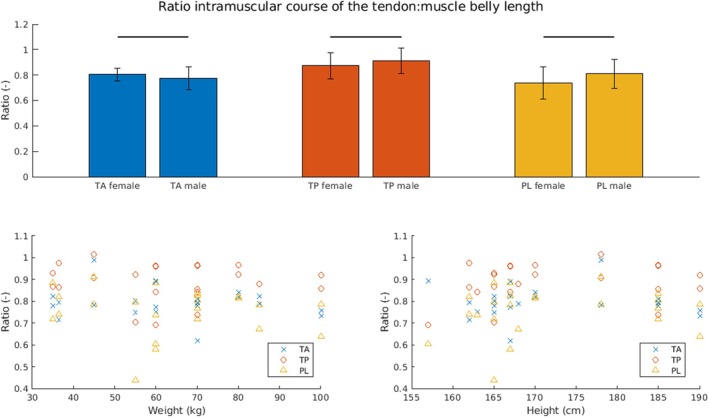
Correlation of the ratio of the intramuscular course of the tendon versus the muscle belly length to donor weight and height. Top row: Ratio of the intramuscular course of the tendon: muscle belly length for females and males. Bottom row: Ratio of the intramuscular course of the tendon: muscle belly length over donor weight (left) and donor height (right). There are no significant correlations between the ratio of the intramuscular course of the tendon: muscle belly length and cadaver donors body weight or height. PL, peroneus longus; TA, tibialis anterior; TP, tibialis posterior.

The MBL of PL and MG demonstrated a significant positive correlation with both donor weight and height (PL weight: correlation coefficient *ρ* = 0.55, *p* < 0.01; PL height: *ρ* = 0.53, *p* < 0.01; MG weight: *ρ* = 0.52, *p* = 0.01; MG height: *ρ* = 0.59, *p* < 0.01) (Figure 5). The TMW of all muscles with the exception of the TP was significantly positively related to both donor weight and height (TA weight: *ρ* = 0.55, *p* < 0.01; TA height: *ρ* = 0.49, *p* = 0.02; PL weight: *ρ* = 0.63, *p* < 0.01; PL height: *ρ* = 0.58, *p* < 0.01; GC weight: *ρ* = 0.86, *p* < 0.01; GC height, *ρ* = 0.52, *p* = 0.01) (Figure 6). Interestingly, there were no significant correlations between the ratio of the intramuscular course of the tendon: muscle belly length and donor weight or height (Figure 7).

## 4 DISCUSSION

The incorporation of AMI construction into transtibial level amputation procedures has been associated with a host of benefits compared with standard approaches to transtibial amputation, including enhanced functional, morphological, and sensorineural outcomes (Berger et al., 2023; Chiao et al., 2023; Chicos et al., 2023; Clites et al., 2019; Clites, Herr, et al., 2018). The presumptive source of these benefits has been the preservation of proprioceptive signaling pathways between the residual limb and the central nervous system enabled by the activation of native mechanoreceptors present in the substance of the coapted muscle–tendon units. The extent to which such mechanoreceptors remain present within AMI components has come into question, however, due to the fact that the muscles utilized in AMI construction in a transtibial amputation scenario must often be shortened proximal to their visible musculotendinous junction prior to coaptation. Until now, the implicit assumption in AMI construction has been that sufficient intramuscular tendon substance exists to result in preservation of a reservoir of MS and GTOs capable of stimulation with AMI activation.

This study has served to validate this assumption—specifically, it revealed that solid tendinous structures could still be found throughout the majority of muscles traditionally utilized for AMI construction in a transtibial amputation scenario. Of the four muscles typically recruited in AMI construction at the transtibial level, two (TA and TP) were noted to have intramuscular tendon extending over 75% of their muscle belly length, one (PL) was noted to have it nearly 90% of its length and one (LG) was noted to have it 100% of its length (i.e., up to its origin). Given that AMI construction in a transtibial scenario is generally performed at the junction of the proximal and middle third of the leg, sufficient length of intramuscular tendon within AMI component musculature is essentially assured with standard AMI design. Furthermore, this study suggests that even proximal level amputations should still permit the construction of AMIs that would include ample numbers of classical proprioceptors such as GTOs and MS.

It should be noted that in the typical transtibial level amputation incorporating AMI construction, not all AMI component muscles are shortened proximal to their visible musculotendinous junction. The LG, for example, is generally routed posterior to the other muscles of the superficial posterior compartment and coapted to the residual TA via its tendinous contribution to the Achilles; as such, concerns about exclusion of the tendinous portion of this AMI component generally do not apply (Berger et al., 2023; Chiao et al., 2023; Clites, Herr, et al., 2018; Herr et al., 2021). Should sufficient length of the LG not be present for AMI construction, however, this study suggests that the MG could be recruited in its place and would provide an adequate reservoir of proprioceptive mechanoreceptors despite the need to shorten it proximal to its visible musculotendinous junction.

One weakness of this study is that it assumes that the density of MS and GTOs in muscle–tendon units proximal to the visible musculotendinous juncture is constant (or at least sufficiently robust to compare favorably with fully intact muscle–tendon units). Understanding the distribution of GTOs and MS within the extremities' muscle bellies is especially of interest to identify whether proximal level amputations or revision scenarios where the distal tendons have been transected would be amenable to AMI construction.

Our investigation did not include a histological assessment of the intramuscular tendon components, nor of the proximal muscle bellies themselves. However, previously published studies related to the microscopic assessment of muscle anatomy have shown that the expression and topography of MS and GTOs in skeletal muscles vary widely in mammals and are not limited solely to the musculotendinous junction (Blumer et al., 2023; Han et al., 2020; Jami, 1992). In extraocular muscles of ungulates, the distal tendon extends onto the muscle surface and GTOs were found in the intramuscular course of the tendon parallel to the muscle fibers (Blumer et al., 2003; Blumer et al., 2006; Blumer et al., 2023). Further histological investigation of receptor density specific to the muscle configurations described in this study may be warranted.

## 5 CONCLUSION

AMI construction requires the incorporation of viable proprioceptive mechanoreceptors such as MS and GTOs into its muscle components to recapitulate the neural pathways that characterize limb position sense in an uninjured state. It is believed that preservation of tendon substance is critical to maintaining an appropriate reservoir of such mechanoreceptors. This study validates the assumption that the intramuscular tendon portion of each muscle traditionally recruited for AMI construction in a transtibial amputation scenario extends sufficiently proximal to ensure its robust inclusion in AMI design. It furthermore provides an anatomic justification for effective employment of leg muscle–tendon unit utilization in AMI construction in more proximal transtibial level amputation scenarios and residual limb revision scenarios.

## CONFLICT OF INTEREST STATEMENT

The authors report no conflicts of interest in this work.

## ETHICS STATEMENT

All investigations were carried out on human body donors who had voluntarily bequeathed their bodies for medical education and research to our institute at Hannover Medical School (MHH) during their lifetimes, after informed consent, in accordance with ethical guidelines. The authors made every effort to follow all local and international ethical guidelines and laws that pertain to the use of human cadaver donors in anatomical research (Iwanaga et al., 2022).

## PATIENT CONSENT STATEMENT

The patients gave their written lifetime approval to make their bodies available for research and education after death.

## Supporting information


Figure S1.

Figure S2.

Figure S3.

Figure S4.

Figure S5.

Figure S6.

Figure S7.

Figure S8.

Figure S9.

Figure S10.

Figure S11.

Figure S12.

Figure S13.

Figure S14.

Figure S15.

Figure S16.

Figure S17.

Figure S18.

## Data Availability

Datasets are available on request.
