# Supplementary figures and images for: Intramuscular tendon length in agonist–antagonist myoneural interface components in transtibial amputation: An anatomic study
Source: J Anat. 2025 May 9;248(4):644–52. doi: 10.1111/joa.14250 (PMC13140445; doi:10.1111/joa.14250)

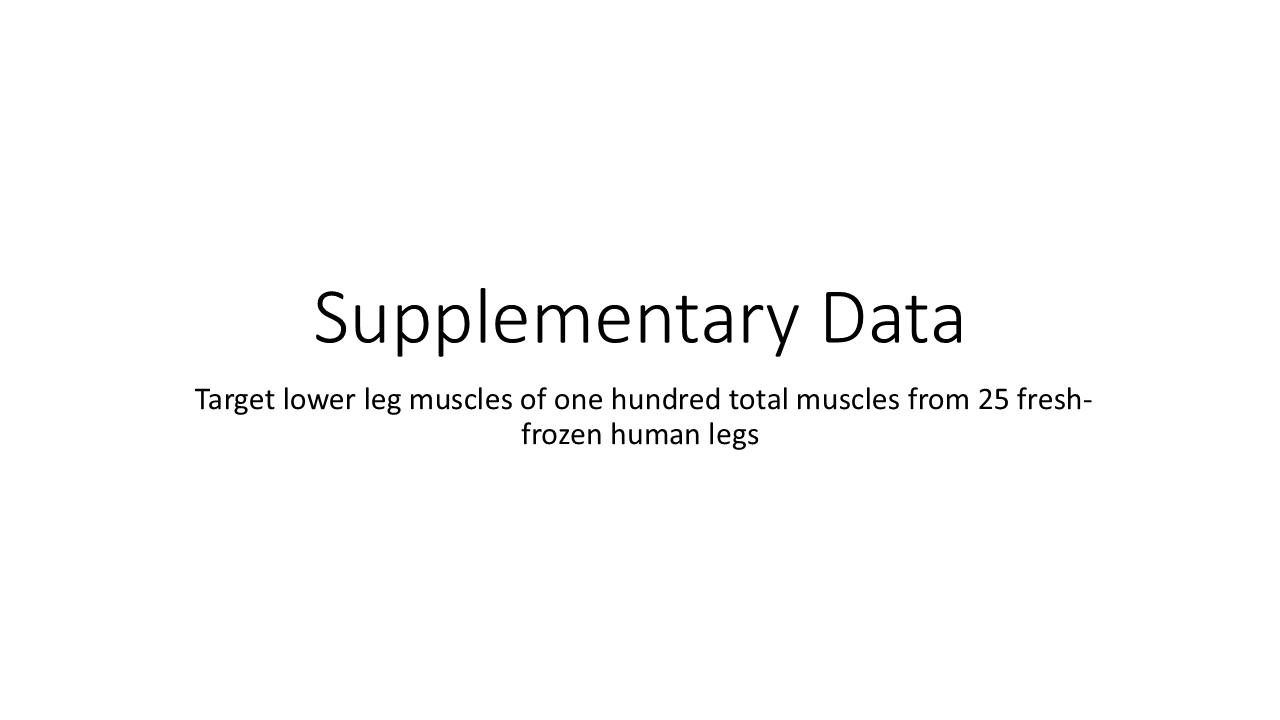

Supplement: Supplementary file 1 — Figure S1. [file JOA-248-644-s017.tif]

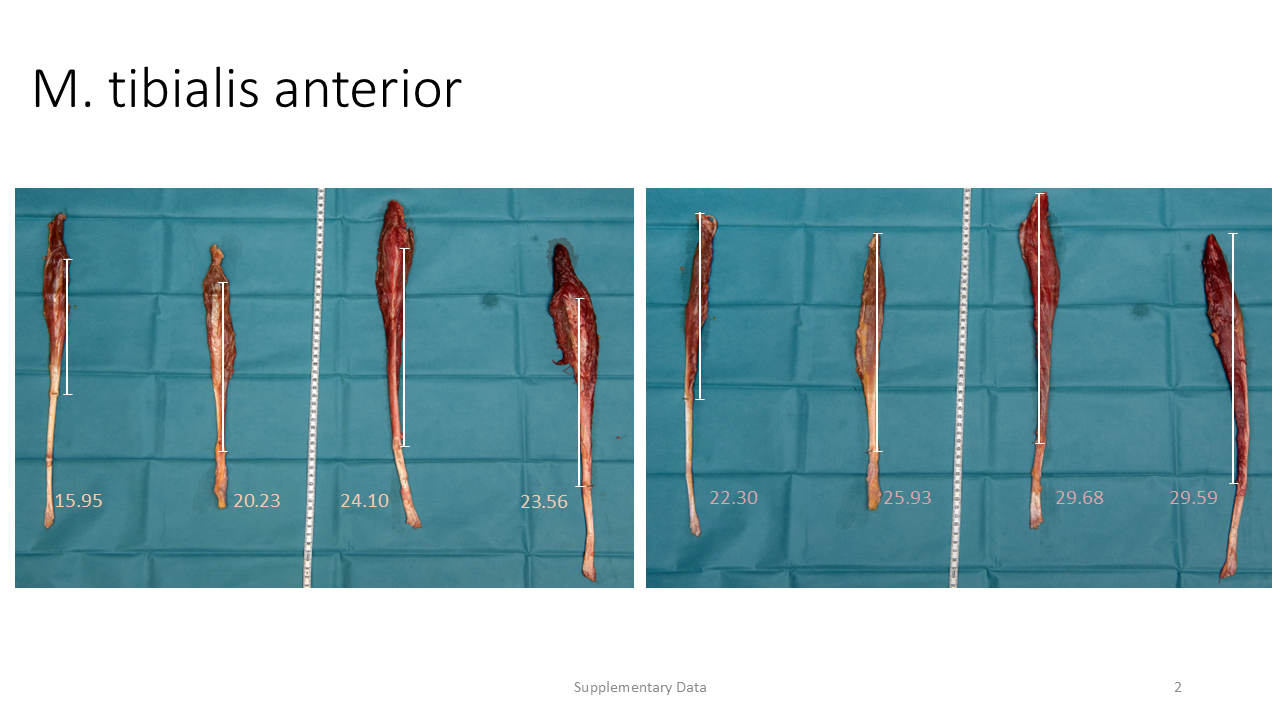

Supplement: Supplementary file 2 — Figure S2. [file JOA-248-644-s014.tif]

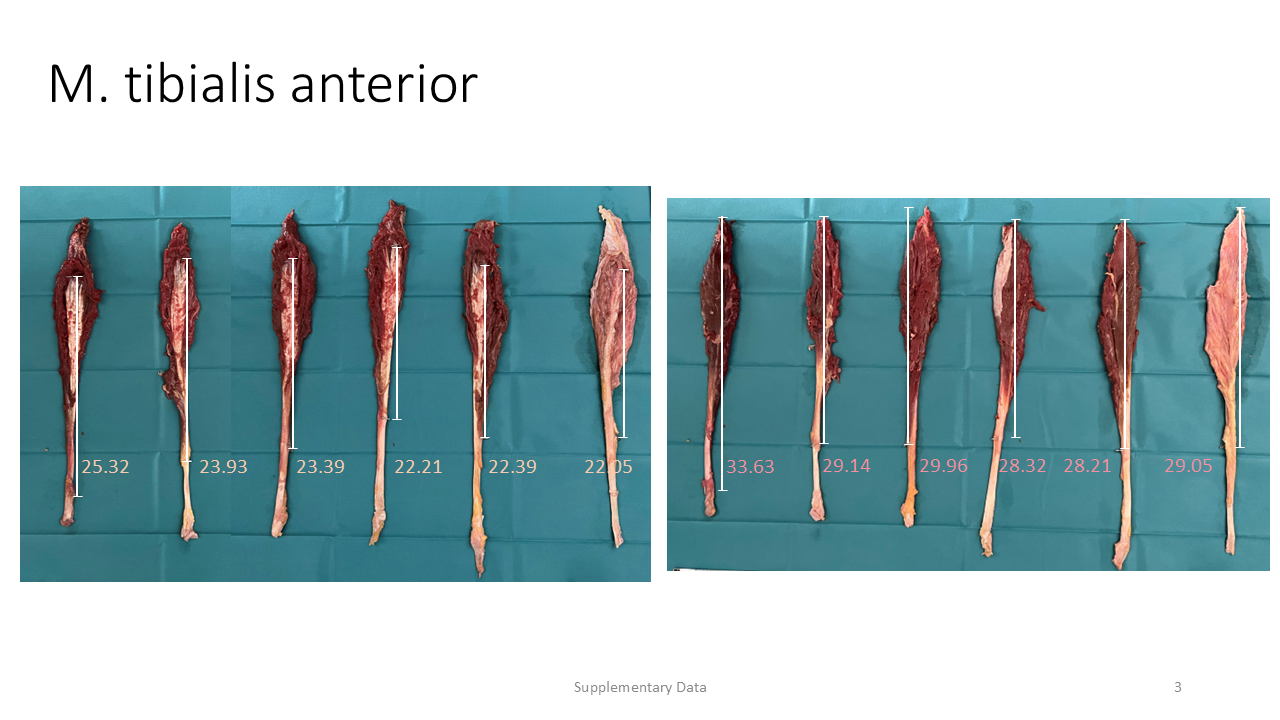

Supplement: Supplementary file 3 — Figure S3. [file JOA-248-644-s002.tif]

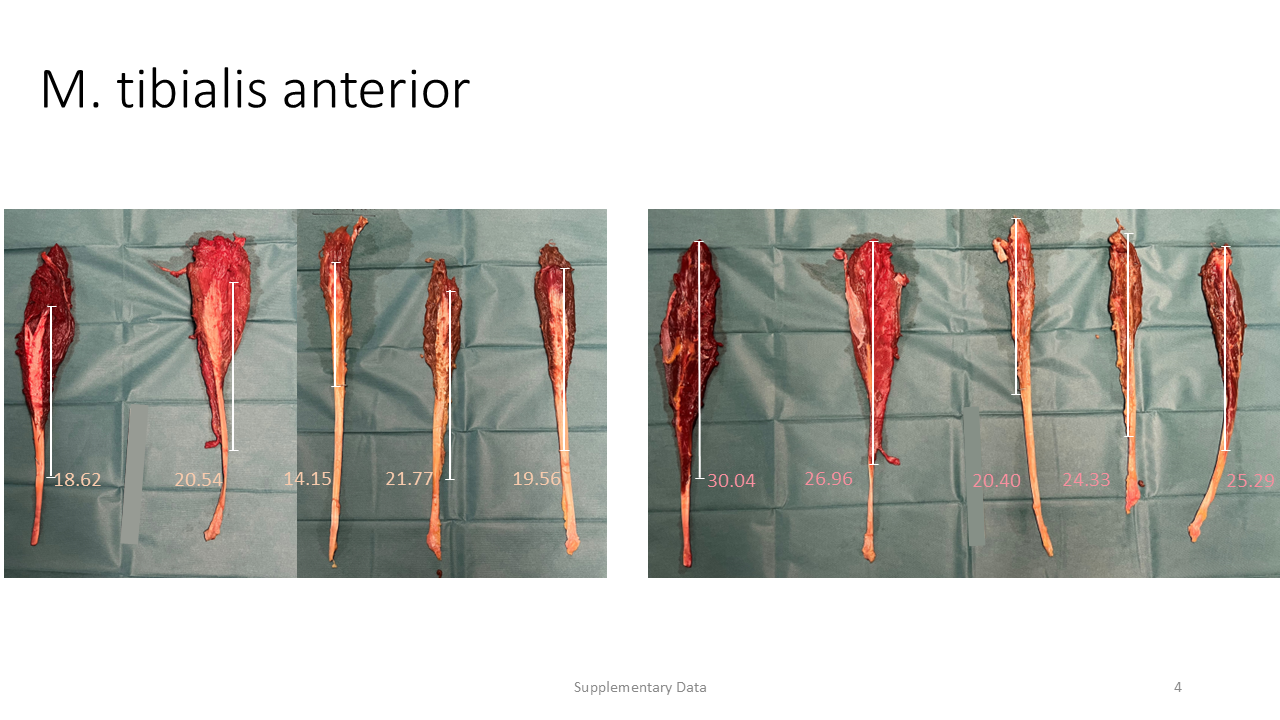

Supplement: Supplementary file 4 — Figure S4. [file JOA-248-644-s013.tif]

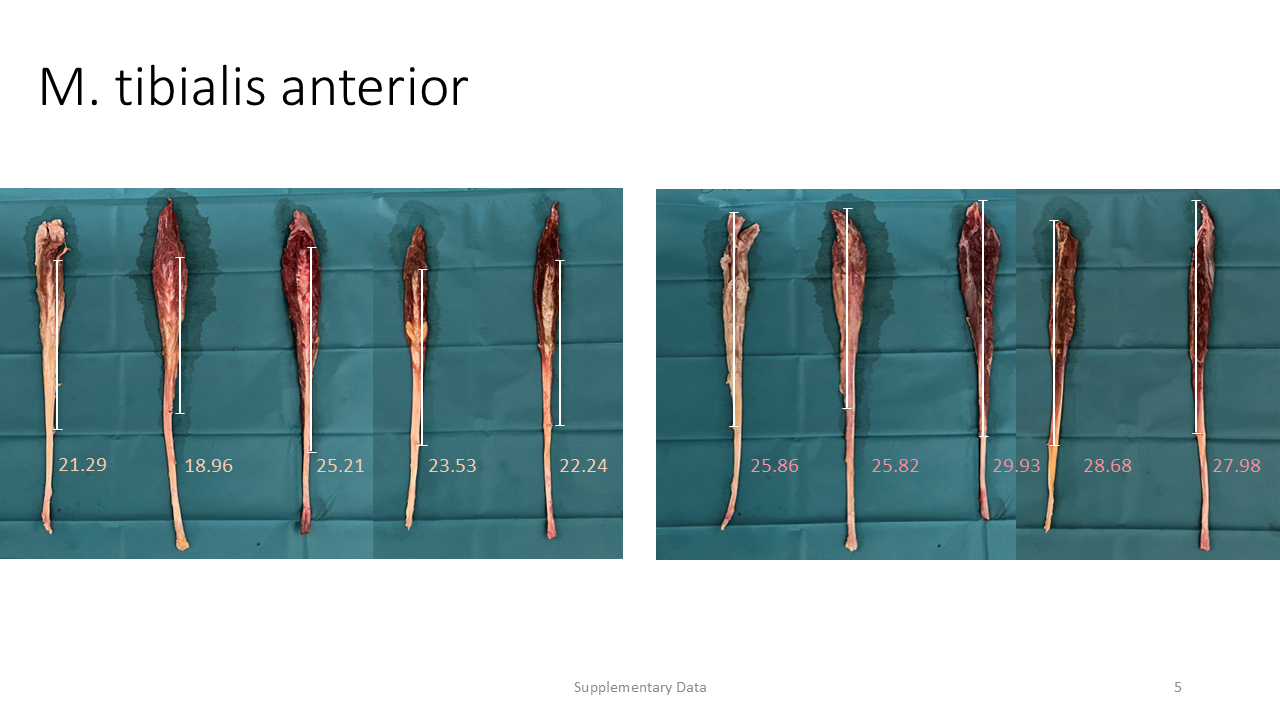

Supplement: Supplementary file 5 — Figure S5. [file JOA-248-644-s007.tif]

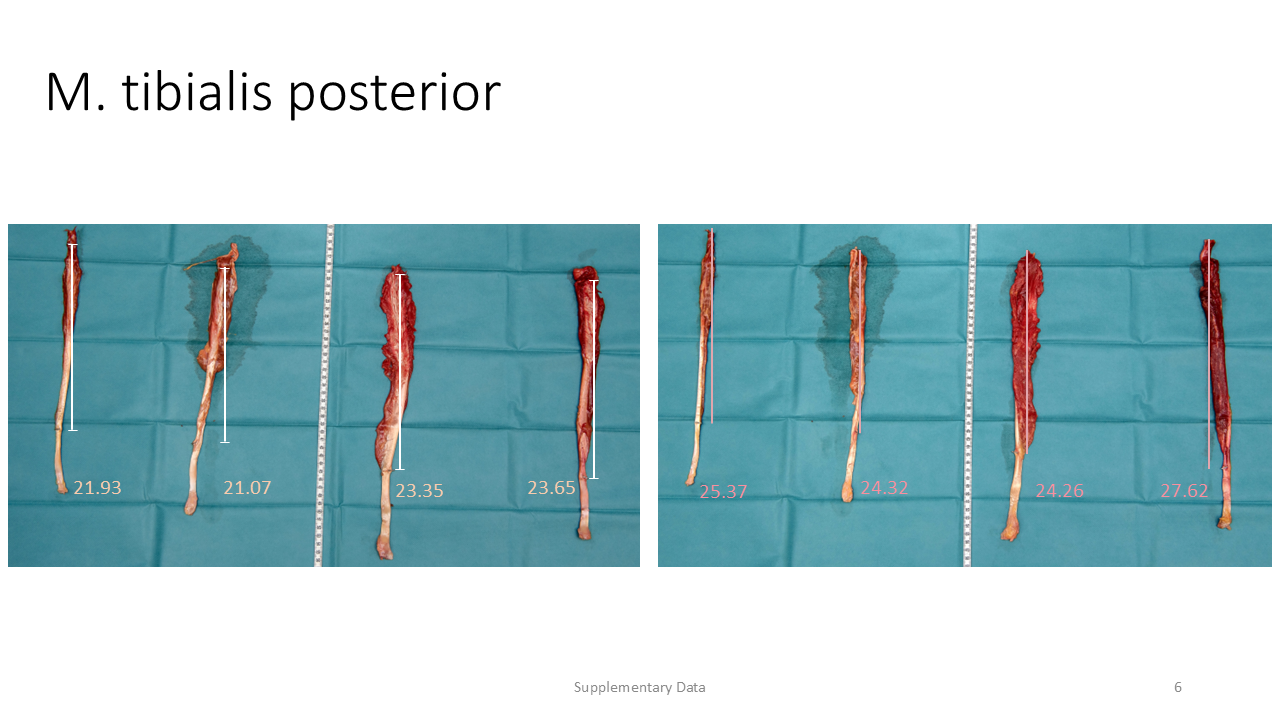

Supplement: Supplementary file 6 — Figure S6. [file JOA-248-644-s006.tif]

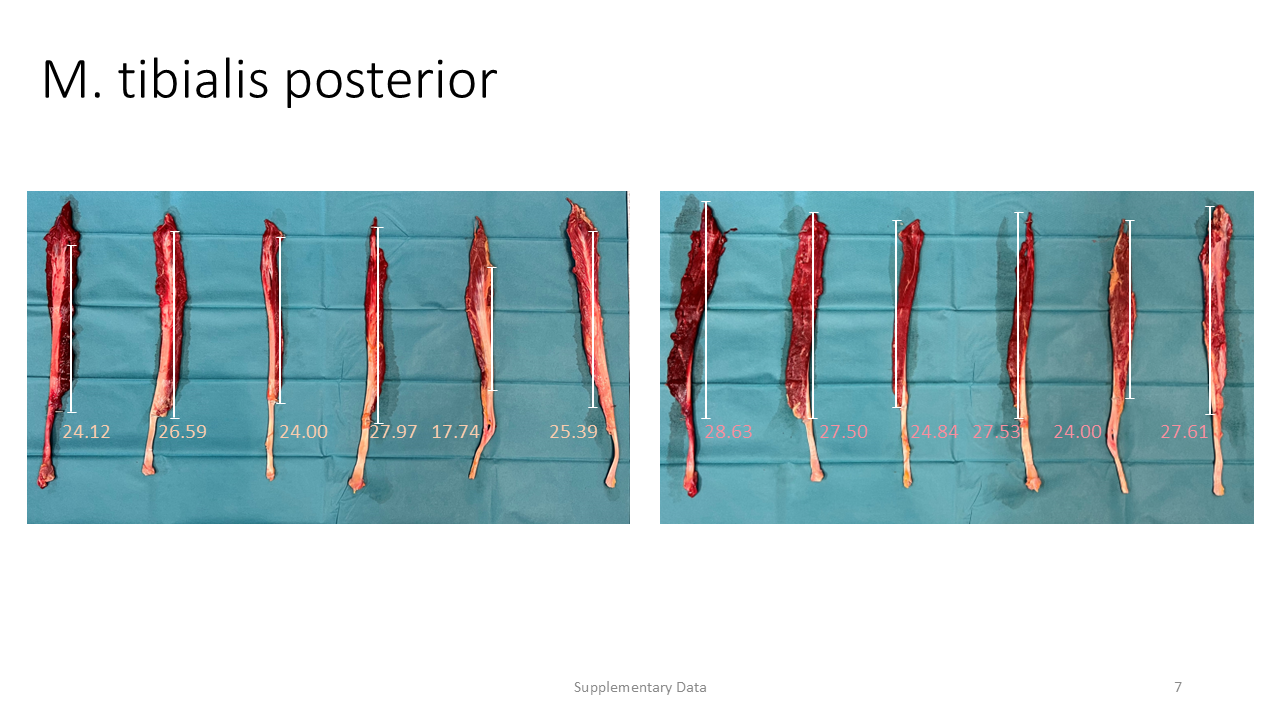

Supplement: Supplementary file 7 — Figure S7. [file JOA-248-644-s012.tif]

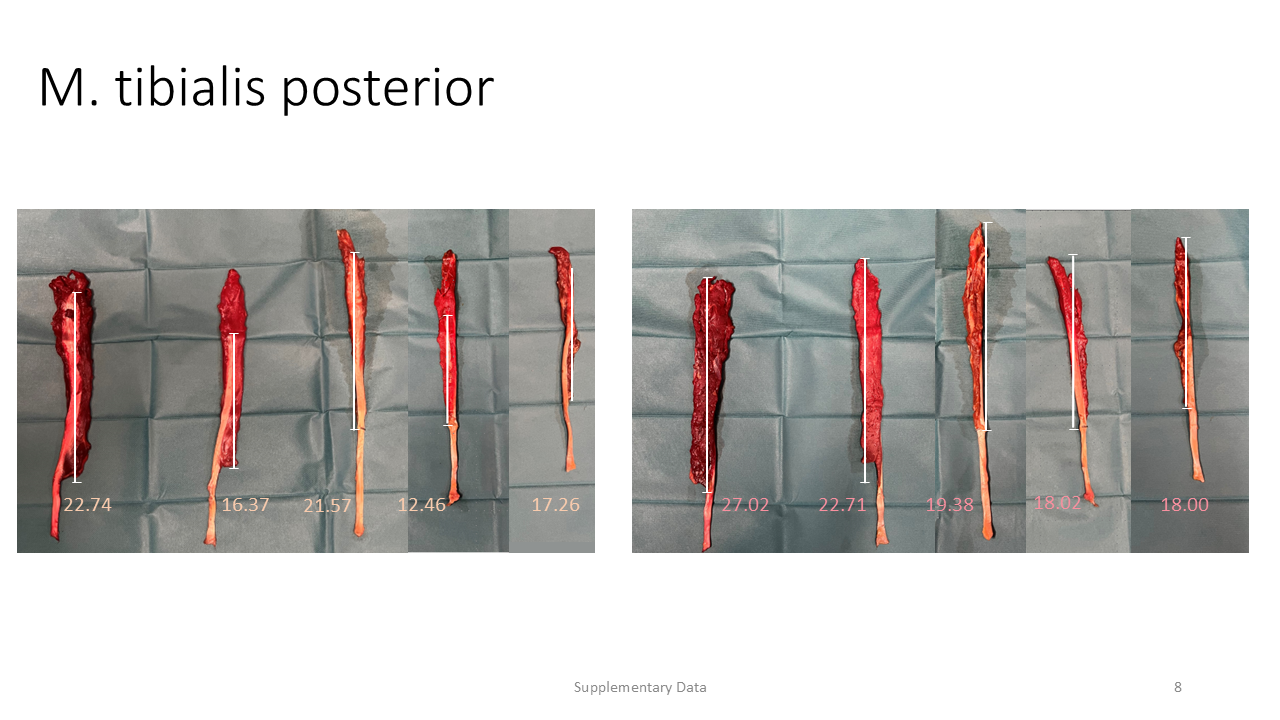

Supplement: Supplementary file 8 — Figure S8. [file JOA-248-644-s004.tif]

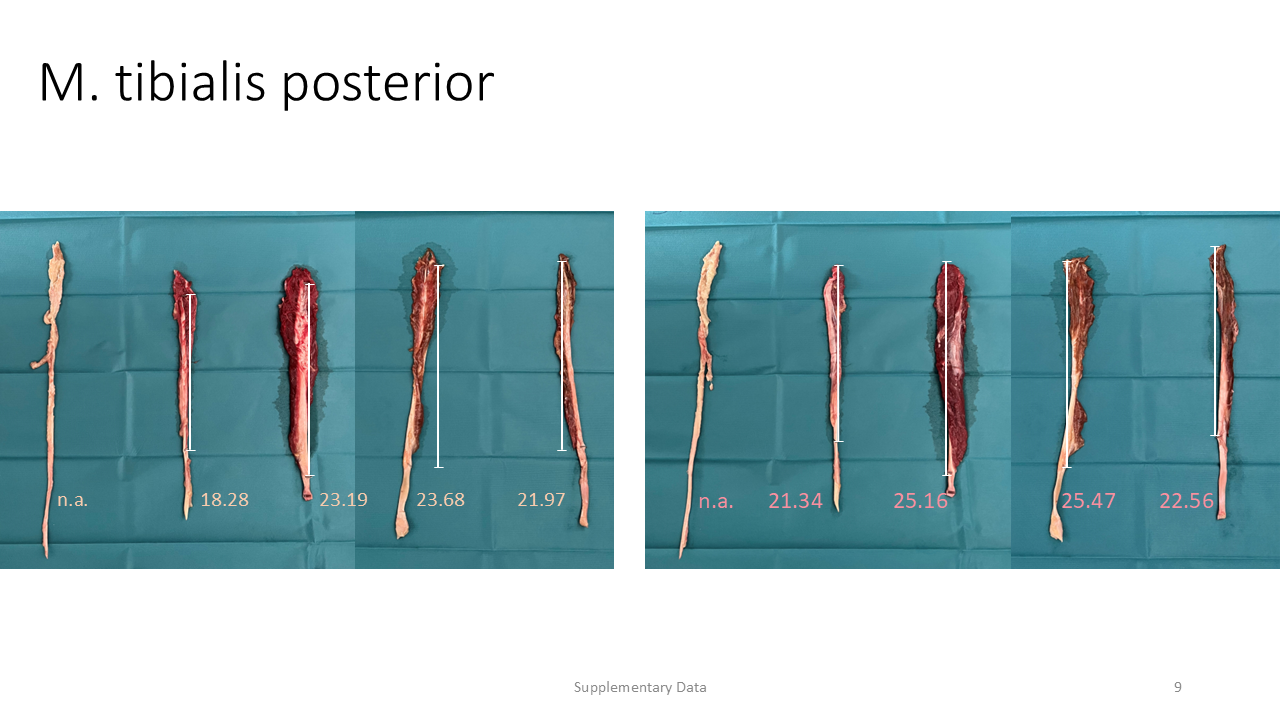

Supplement: Supplementary file 9 — Figure S9. [file JOA-248-644-s016.tif]

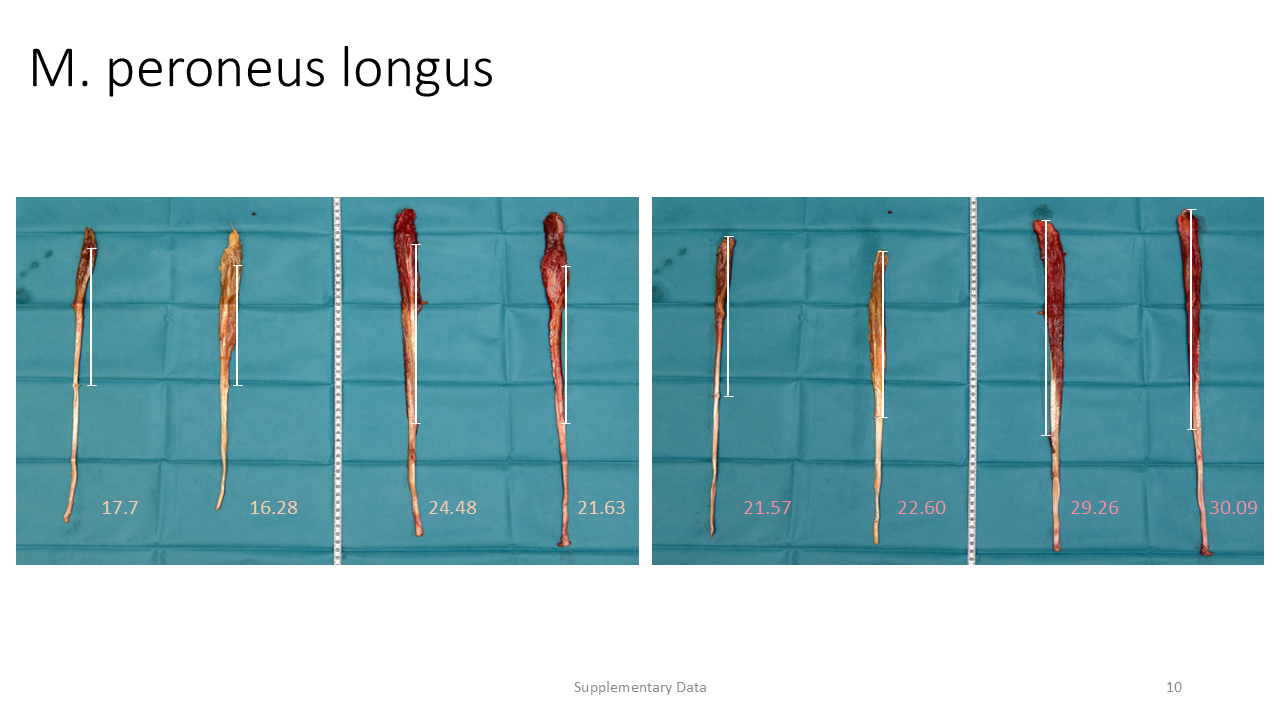

Supplement: Supplementary file 10 — Figure S10. [file JOA-248-644-s011.tif]

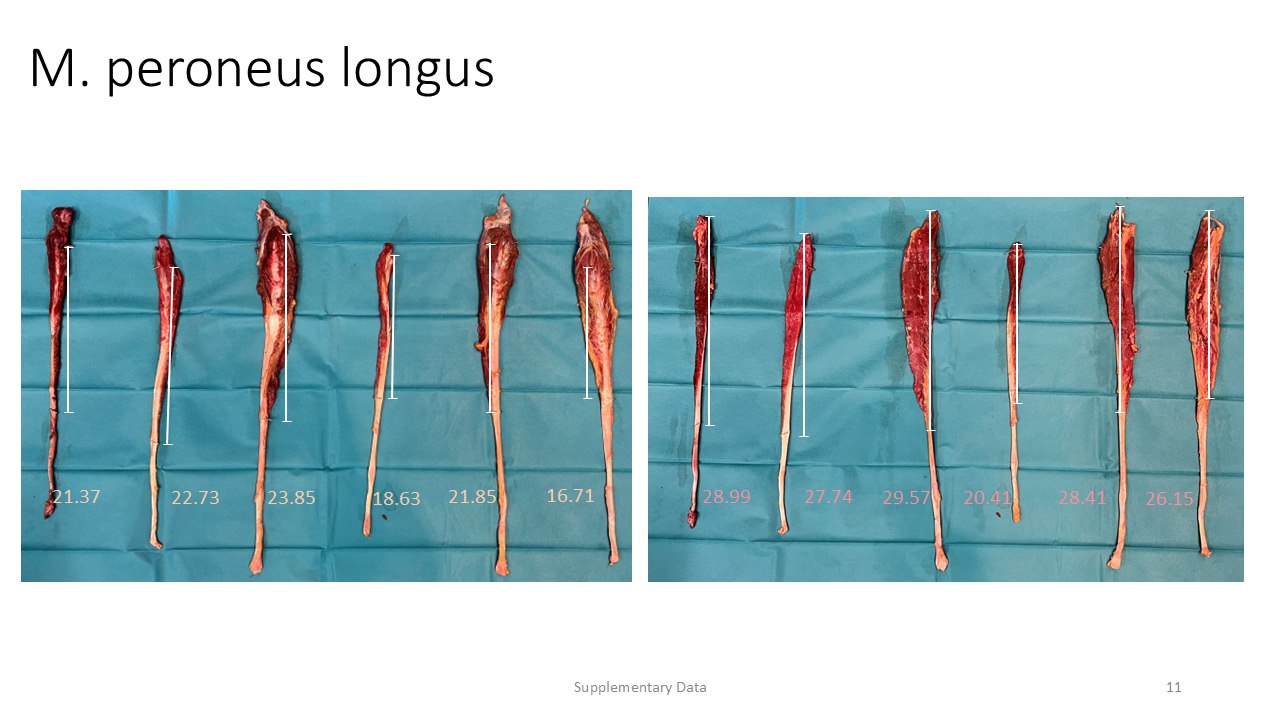

Supplement: Supplementary file 11 — Figure S11. [file JOA-248-644-s008.tif]

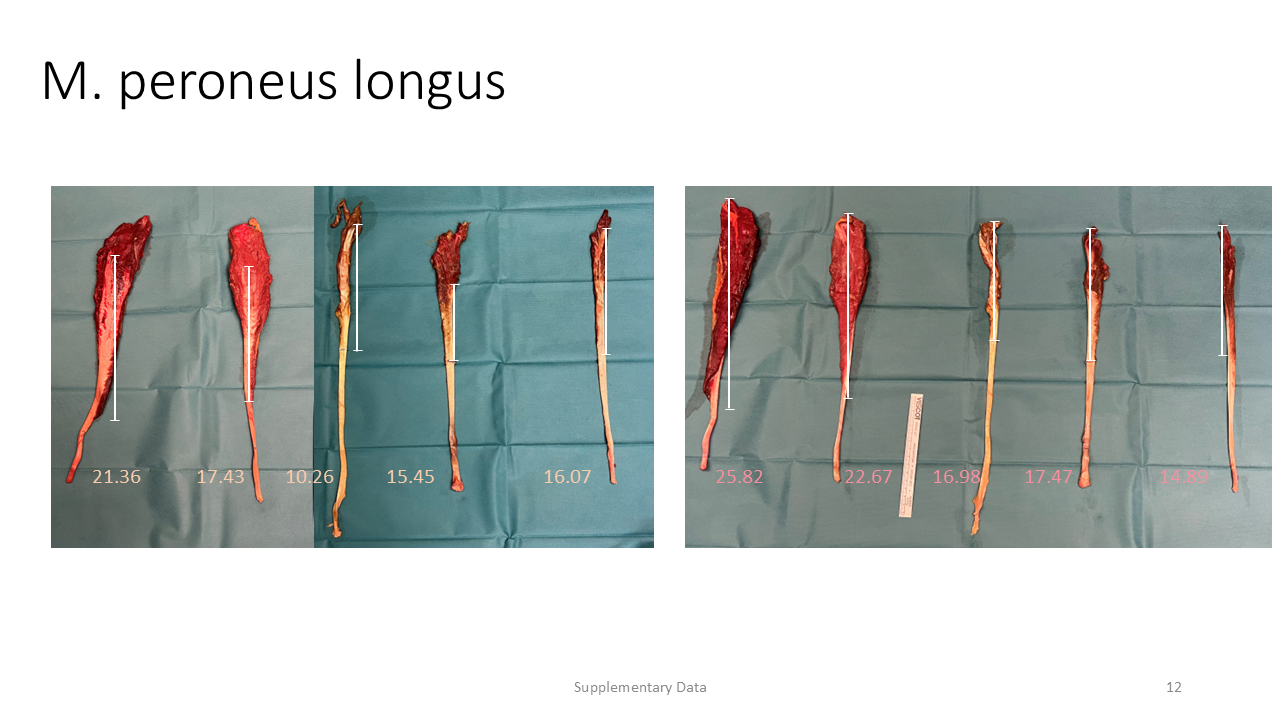

Supplement: Supplementary file 12 — Figure S12. [file JOA-248-644-s003.tif]

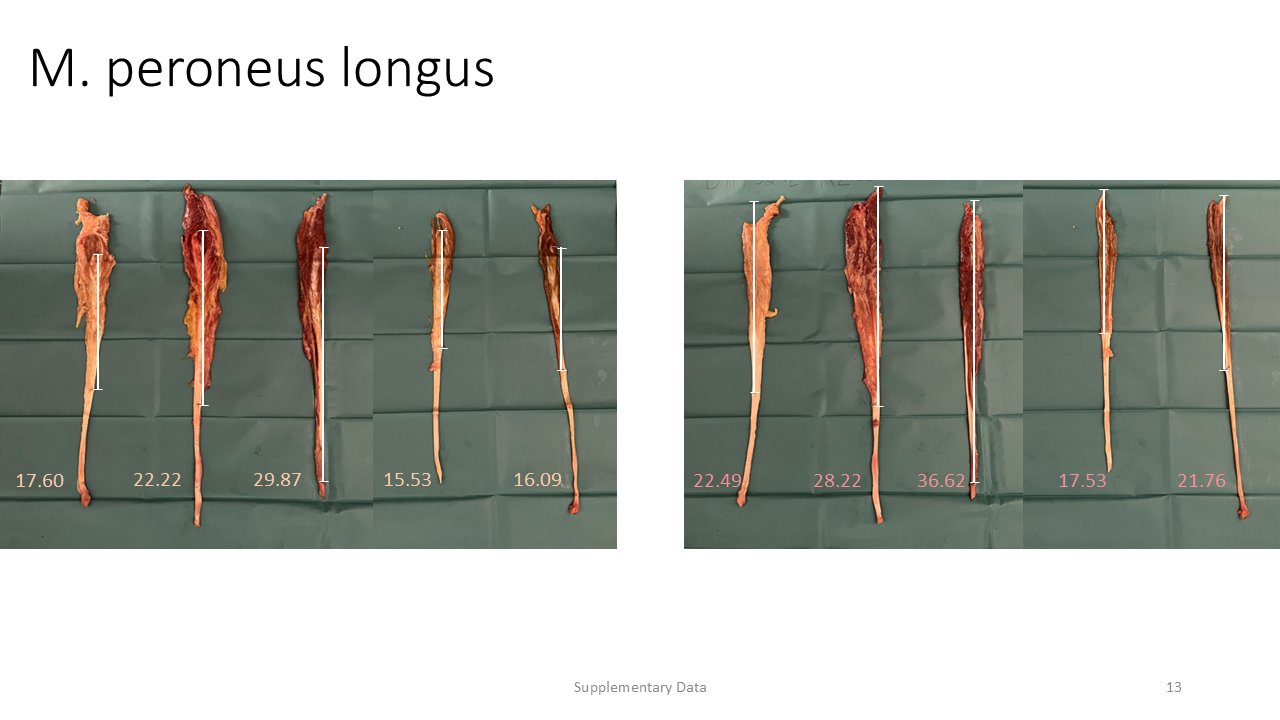

Supplement: Supplementary file 13 — Figure S13. [file JOA-248-644-s015.tif]

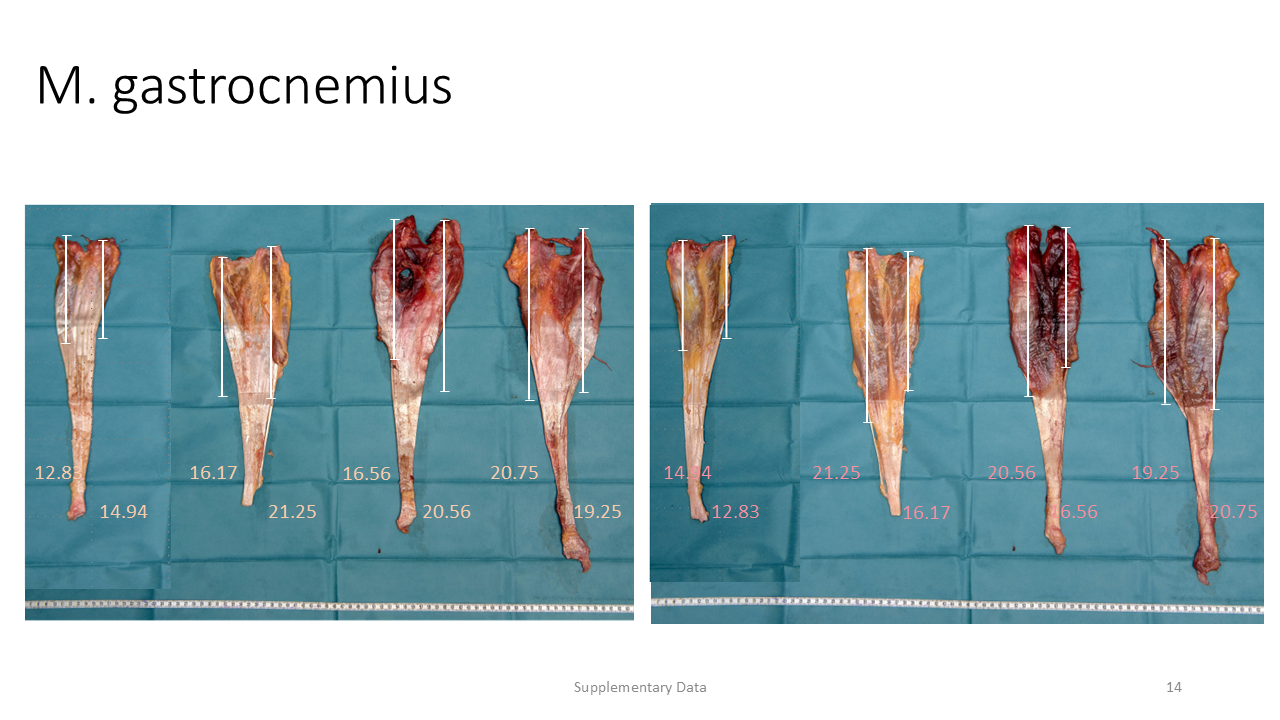

Supplement: Supplementary file 14 — Figure S14. [file JOA-248-644-s018.tif]

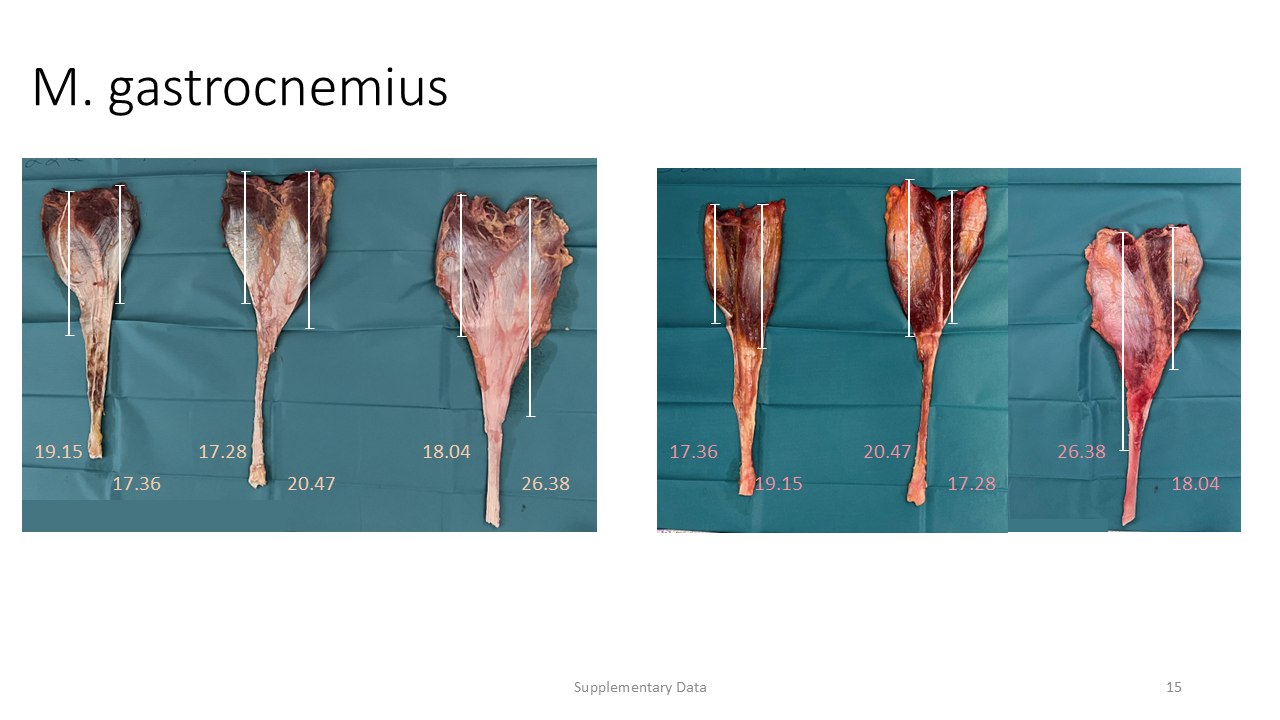

Supplement: Supplementary file 15 — Figure S15. [file JOA-248-644-s001.tif]

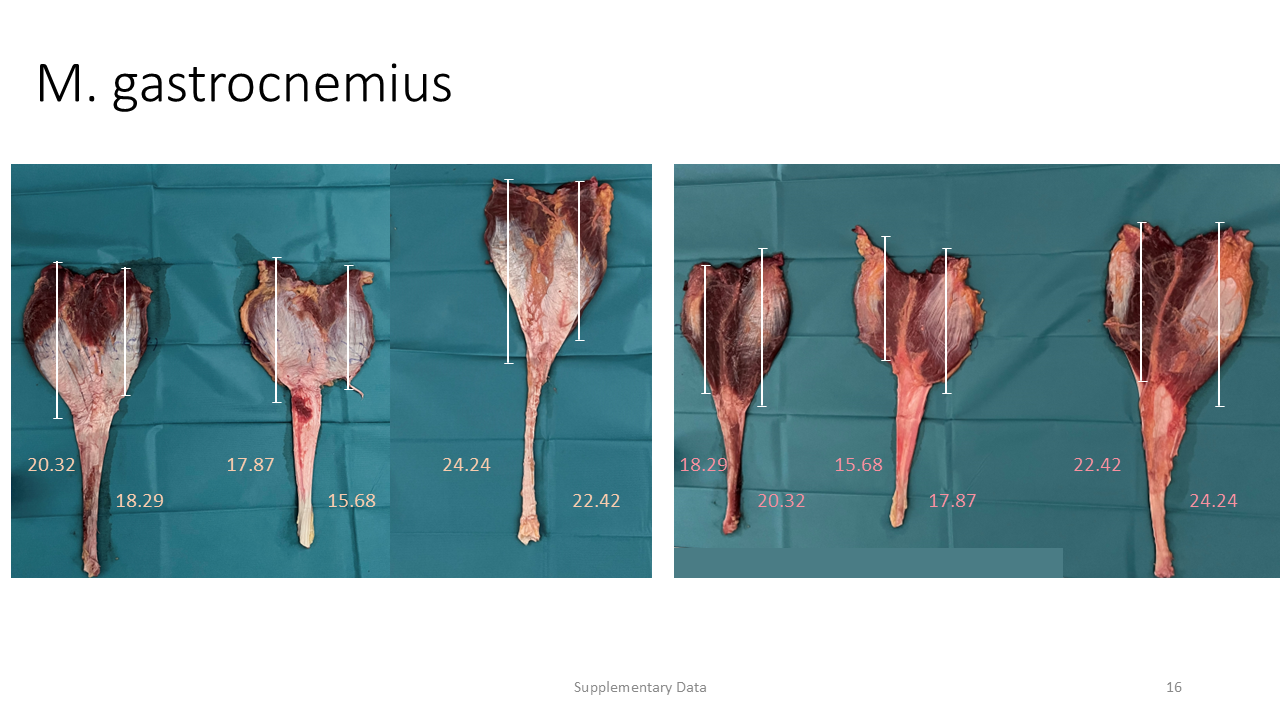

Supplement: Supplementary file 16 — Figure S16. [file JOA-248-644-s005.tif]

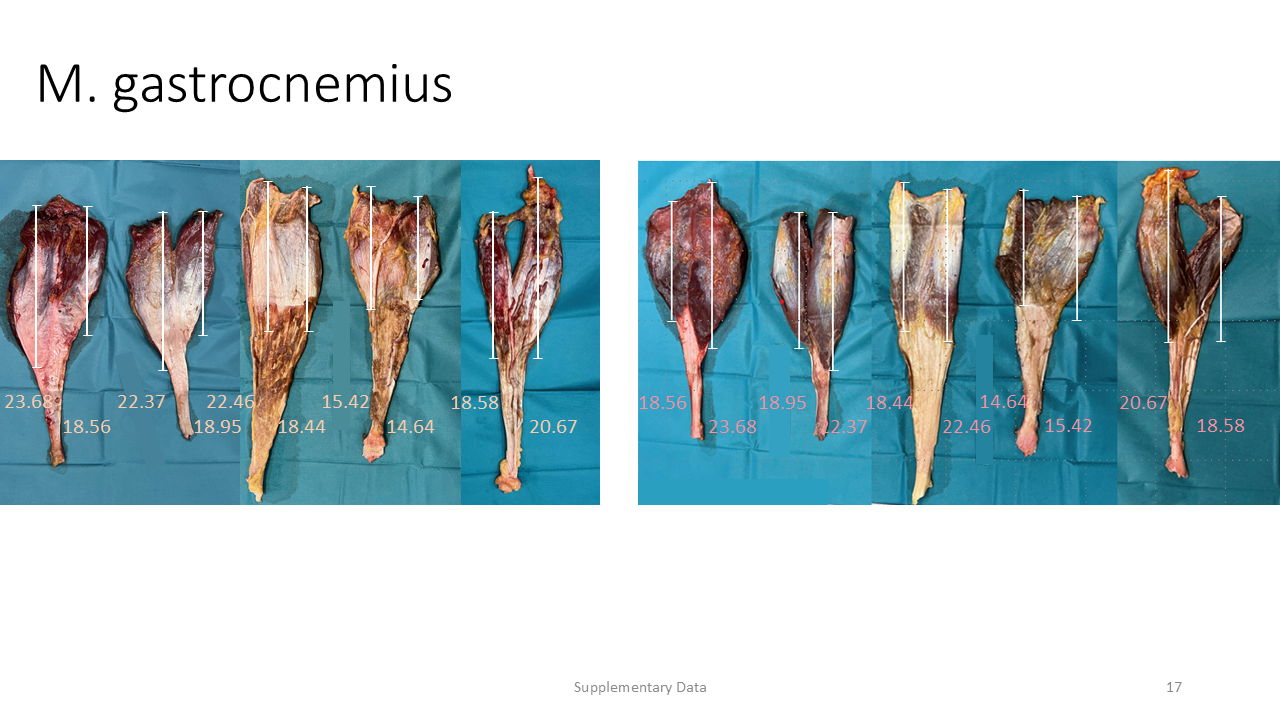

Supplement: Supplementary file 17 — Figure S17. [file JOA-248-644-s009.tif]

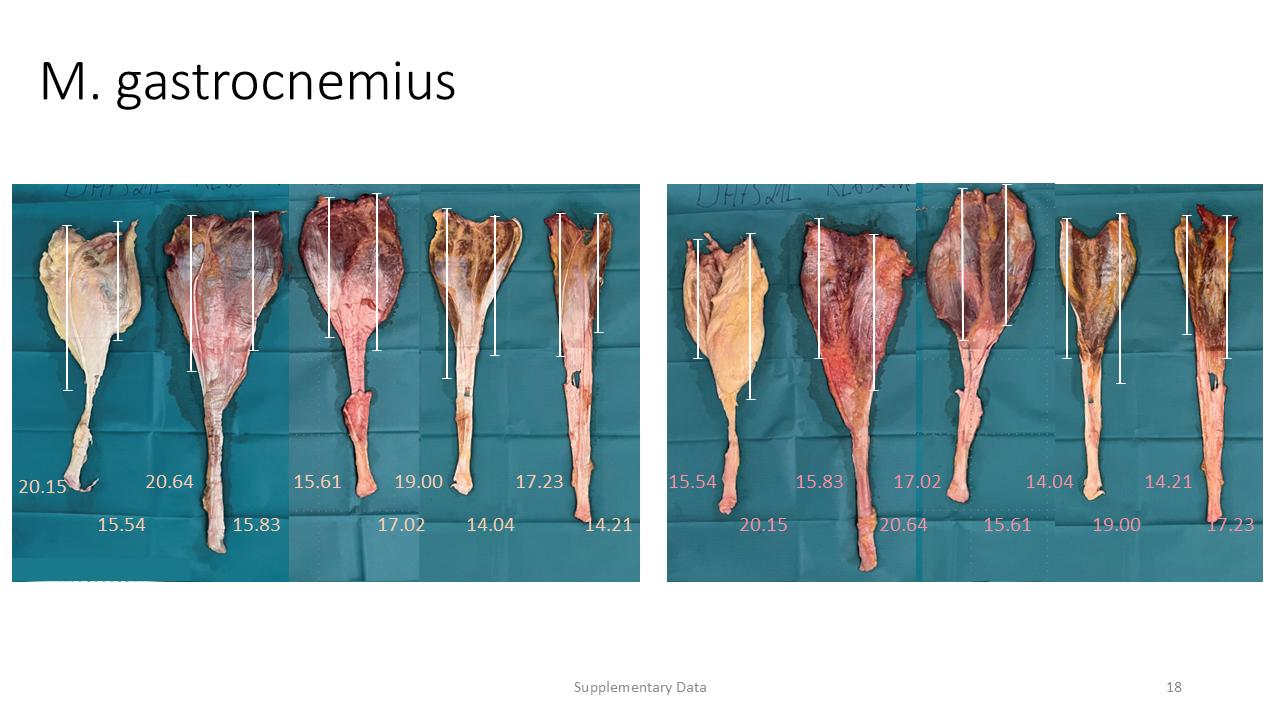

Supplement: Supplementary file 18 — Figure S18. [file JOA-248-644-s010.tif]
